# Diagnostic delay in patients with sporadic hereditary transthyretin-mediated amyloidosis

**DOI:** 10.1007/s10072-024-07793-1

**Published:** 2024-10-10

**Authors:** Bernardo Antunes, Isabel Conceição, Catarina Falcão de Campos, Mamede de Carvalho

**Affiliations:** 1 Instituto de Fisiologia, Centro de Estudos Egas Moniz, Faculdade de Medicina, Universidade de Lisboa, Lisbon, Portugal; 2 Departamento de Neurociências e Saúde Mental, ULS de Santa Maria, Centro Académico de Medicina de Lisboa, Lisbon, Portugal

**Keywords:** ATTRv amyloidosis, Diagnostic delay, Familial amyloid polyneuropathy, Misdiagnosis, Sporadic cases

## Abstract

**Background:**

Hereditary transthyretin-mediated amyloidosis (ATTRv amyloidosis) is a rare progressively incapacitating condition with a wide range of genotype/phenotype presentations. It is frequently diagnosed late in its course, particularly in sporadic cases.

**Objectives:**

Analysing predictors of diagnostic delay in this subpopulation should be, therefore, a priority.

**Methods:**

109 apparently sporadic ATTRv amyloidosis patients followed in a reference centre in Hospital de Santa Maria (ULS Santa Maria-CAML), in Lisbon, were studied. Time from symptom onset to diagnosis, age, sex, municipality of origin and initial symptoms were obtained. Diagnostic delay was compared between different decades with a Kruskal–Wallis test, and its predictors were evaluated in a univariate model followed by a binary logistic regression analysis to calculate the adjusted odds ratio.

**Results:**

The median diagnostic delay was 1262 days. There was a non-significant difference in diagnostic delay between the 80 s, 90 s, 2000s and 2010s decades. There was a non-significant trend for a longer diagnostic delay in woman and in patients having no neurologic symptoms at onset.

**Conclusion:**

There is an important diagnostic delay in sporadic cases of ATTRv amyloidosis. Awareness should be spread among clinicians regarding the various manifestations of this disease, stressing the importance of family history and epidemiological data.

## Introduction

Hereditary transthyretin-mediated amyloidosis (ATTRv amyloidosis) is a rare progressively incapacitating multisystemic disorder [1]. The majority of cases manifest as either predominantly neuropathic (ATTRv polyneuropathy), primarily cardiac (ATTRv cardiomyopathy), or as a mixed phenotype [1]. ATTRv polyneuropathy is characterized by a length-dependent polyneuropathy with autonomic dysfunction [2]. The disease is now known to be spread worldwide, having its biggest prevalence in Portugal, where the threshold for rare disease (“any disease affecting 5 or less in 10000 persons in the community”) is exceeded in 19 out of the 174 municipalities where there are cases of ATTRv amyloidosis [3].

Survival from symptom onset ranges from 6 to 12 years [4], depending on various variables such as age of onset and phenotype, with known cardiac involvement associated with shorter survival [5–7].

Before 1990 only symptomatic management was available, but nowadays we have disease-modifying therapies (DMT) approved for effectively halting disease progression and extending survival [8]. Despite important advancements, treatment success rate is greater when given in early stages [8, 9]; however, the disease is still frequently diagnosed late in its course [4]. The longer the delay, the greater the damage in multiple organs due to increasing amyloid burden [10], compromising the success of the current treatments [8, 9]. The early identification of the disease can also potentiality lead to prompt genetic screening in family members, thus allowing the initiation of an appropriate follow-up [11–13].

It is estimated that 32–74% of patients are misdiagnosed [14, 15] and 18% have multiple incorrect diagnoses before the proper one is established [15]. Factors such as the diversity of clinical presentation [16] and its low prevalence [17] make diagnosis harder for health care professionals not familiar with this disease. Besides, and of great importance, being a hereditary condition with incomplete penetrance, which varies in different geographical regions [18, 19], the delay in diagnosis is particularly a problem for patients without a positive family history of ATTRv amyloidosis [10, 20, 21].

Our research, therefore, targets this specific population of patients without familial history, aiming to dissect the diagnostic delay and its change over different decades. In particular, intends to identify clinical predictors of diagnostic delay. This is imperative to introduce changes for promoting earlier medical intervention.

## Material and methods

### Study design and population

A retrospective study was conducted in a large population of ATTRv amyloidosis patients. A data base with a total of 766 patients diagnosed between 1986 and 2021 in the reference centre for hereditary amyloidosis at Hospital de Santa Maria (ULS Santa Maria-CAML), in Lisbon, was used to analyse the data. Clinical diagnosis was supported by the presence of a pathogenic mutation in the TTR gene and amyloid deposits in the tested tissues (more frequently in salivary glands). All patients underwent regular laboratory tests (including renal function markers), neurophysiological evaluations (confirming length-dependent predominant axonal neuropathy), and cardiac assessments throughout the follow-up period. Cardiac evaluations have evolved over time due to technological advancements, now encompassing laboratory tests (NT-proBNP), electrocardiography (Holter monitoring), echocardiography, and “bone” cardiac scintigraphy in all patients.

The population of this study consisted of individuals whose family history of the disease was negative or unknown at the time of diagnosis, comprising 133 patients. This included not only truly sporadic cases, but also those with a probable/confirmed family case identified after the diagnosis had been made. This happened when retrospectively evaluating signs and symptoms of family members, which had not been considered relevant until that moment.

### Exclusion criteria and data imputation

Patients without information on symptoms onset and/or diagnosis, and those missing data on age, sex, municipality of origin and on specific initial symptoms were excluded from the analysis. Whenever there was a doubt regarding diagnostic time, the date of the positive genetic test was considered. Every time the month or day of symptoms onset or diagnosis was missing, the first of July and the 15th of the corresponding month was used to calculate the delay. This was thought to be the best approach to minimize the error associated with the imputation of data.

### Variables

The dependent variable in this study is the diagnostic delay, defined as the time from symptom onset until diagnosis. This was then categorized in “delayed” or “not delayed”. According to our knowledge, there is no reference value in literature for this purpose. Therefore, the 3rd quartile of the recorded values from this population was considered as the cut-off point for defining “delayed” or “not delayed”.

The independent variables in this study, which were also categorized as binary variables, were sex, age at onset, municipality of origin and initial symptoms. For age of symptom onset, a consensual age reported in literature was used, subdividing them into “early onset” (< 50 years) and “late onset” (≥ 50 years) groups [22–24]. Patients were also divided according to “high risk area” and “low risk area” of presenting the disease, to study the influence of the origin of patients. To determine which patients belonged to each category, we considered the fact that, in Portugal, the threshold for rare disease is exceeded in 19 municipalities [3]. Patients coming from these municipalities were included in the “high risk area” category, while all others were included in the “low risk area” category. As for initial symptoms, patients were subdivided into a group having “neurologic manifestations” and a group with “no neurologic manifestations”. Patients who had signs and symptoms of polyneuropathy, autonomic dysfunction (including gastrointestinal disturbances), and/or carpal tunnel syndrome (CTS) were included in the first group. Patients with cardiovascular, renal, ocular and/or isolated weight loss were categorized into the non-neurologic category. According to the decades in which patients began having symptoms, four groups were defined: symptom onset in the 80 s, 90 s, 2000s and 2010s.

### Statistical analysis

The Kruskal–Wallis test (data with non-normal distribution, Shapiro–Wilk Test) was used to test significant differences in the diagnostic delay through the various decades. Chi-Square Test or Fisher’s Exact test were applied to check if there was an association between the independent variables (age at onset, sex, municipality of origin and initial symptoms) and the dependent variable (diagnostic delay). Variables that showed marginal associations (p < 0.20) in the univariate analysis were included in a binary logistic regression model, allowing the calculation of the adjusted Odds Ratio and examine whether the analysed factors were independently associated with diagnostic delay. The assumption that there was no multicollinearity was met and an inspection of standardised residual values revealed no outliers. The quality of the adjustment was calculated through the Hosmer and Lemeshow test. It was adopted a level of significance of 0.05. Data was analysed with the 20th version of Statistical Package for Social Science (SPSS).

### Ethics

This study was approved by the CAML Ethics committee (Ref 1117/13).

## Results

After applying the exclusion criteria, 109 patients remained out of the initial 133. After diagnosis, it was found that in 24 patients (approximately 22%) other family members had symptoms compatible with ATTRv amyloidosis. The median disease duration at diagnosis was 3.5 years (1262 days, 1st-3rd IQR 824 and 2007 days respectively), which was not statistically different across the consecutive decades analysed (Table 1).

**Table 1:** Diagnostic delay across different decades

Decade of symptoms onset	Number of cases	Median delay	Kruskall-Wallis Test
	(n)	(days)	(p-value)
80 s	17	1174.0	
90 s	28	1788.5	0.74
2000s	31	1203.0	
2010s	33	1262.0	

The characteristics of the population cohort and the delay in diagnosis associated with each group are presented in Table 2. Most were men, coming from low-risk areas, with late onset symptoms, and with neurologic manifestations at onset. The majority of patients had a Val30Met mutation (105), but 3 had V28M and 2 V122Ile mutations. Gender and symptoms at presentation showed marginal association with diagnostic delay (p < 0.20) – Table 3. Therefore, these two variables were included in the binary logistic regression model. This multivariate analysis disclosed that being a woman and having no neurologic symptoms at onset tended to be predictors of diagnostic delay (adjusted odds ratio of 2.329 and 2.224, respectively), but none of them showed a statistically significant p value (p < 0.05) – Table 4.

**Table 2:** Characteristics of the population and diagnostic delay in different groups

Variables	Total	Mean Delay	Median Delay	1st Quartile	3rd Quartile	IQR^a^
	(n)	(days)	(days)	(days)	(days)	(days)
Gender						
Male	73	1470.4	1232.0	765.5	1863.0	1097.5
Female	36	2067.8	1789.5	924.3	2959.5	2035.3
Age of onset						
Early-onset	30	1507.3	1164.0	741.0	2058.3	1317.3
Late-onset	79	1728.6	1387.0	873.0	2026.0	1153.0
Municipality of origin						
High-risk	26	1441.8	1053.0	722.8	1917.5	1194.8
Low-risk	83	1738.5	1387.0	887.0	2182.0	1295.0
Initial symptoms						
Non-neurologic	15	2245.6	1871.0	1237.0	3310.0	2073.0
Neurologic	94	1575.5	1239.0	742.5	1965.5	1223.0

^a^*IQR* interquartile range

**Table 3:** Factors associated with diagnostic delay

Variables	Total	Delayed^a^	Not delayed	p-value^b^
	(n)	(n)	(n)	
	109	27	82	
Gender				
Male	73	14	59	0.054
Female	36	13	23	
Age of onset				
Early-onset	30	7	23	0.830
Late-onset	79	20	59	
Municipality of origin				
High-risk	26	5	21	0.453
Low-risk	83	22	61	
Initial symptoms				
Non-neurologic	15	6	9	0.195^c^
Neurologic	94	21	73	

^a^As described in methods, diagnostic delay is defined as a time longer that the 3rd quartile of the time distribution; ^b^Chi-Square Test of Independence; ^c^Fisher’s Exact Test

**Table 4:** Binary logistic regression

	B	S.E	Wald	df	*p*	AOR^a^	95% C.I. AOR	
							LL	UL
Gender	0.846	0.461	3.358	1	0.067	2.329	0.943	5.753
Initial Symptoms	0.799	0.594	1.81	1	0.178	2.224	0.694	7.126
Constant	-1.558	0.317	24.149	1	0	0.211	`	

^a^*AOR* adjusted odds ratio

## Discussion

The characteristics of our population are similar to other studies on sporadic cases, with most patients being men, coming from areas where the disease is less prevalent and having a late onset of their symptoms, most of which are neurologic [20, 21]. There was a median delay of 1262 days (~ 3.5 years) from symptom onset to diagnosis, which is close to the delay of 4 years reported by Planté-Bordeneuve et al., [21] in a population of French sporadic cases and 4.5 years reported by Coelho et al. [20], in Portuguese sporadic patients. We consider that this period is far from ideal and should therefore be reduced. According to some sources, the diagnosis of ATTRv amyloidosis can be made within 1 year in endemic areas [1], whereas, in non-endemic areas, there can be a delay of over 3 years [4, 6, 14]. Factors like the existence of a higher prevalence of cases and the possibility for mutation carriers to receive adequate genetic counselling can explain a shorter diagnostic delay in endemic areas [25]. However, even though Portugal holds nearly 20% of ATTRv amyloidosis cases worldwide [3], in our study, the diagnosis in patients without a known family history of the disease was as delayed as in non-endemic areas in general, which highlights the challenges associated with managing these cases, in particular the non-specificity of the initial symptoms originating a wide range of differential diagnosis [26–28].

Despite the amazing evolution in treatment that has been on the rise since the 1990’s [8, 9], which is still most effective early in the course of the disease, we seem to be no better at identifying sporadic cases earlier, since there was no significant difference in the median diagnostic delay of patients whose disease onset occurred in the 80 s, 90 s, 2000s or 2010s. Hence, it is essential that we strive to comprehend what measures can be taken to address this issue.

When analysing the patients included in this study, we did not identify any significant cause for a longer diagnostic delay, which could be due to the relatively small number of patients included; however, there a trend indicating that being woman and having no neurologic manifestations at onset can contribute to a longer diagnostic delay. Regarding gender, these results seem to differ from other studies which included patients with positive and negative familial history, in which being a man was recognized as a factor contributing to misdiagnosis [15]. However, the results in our study may reflect the gender differences in clinical manifestations, in particular the role of erectile dysfunction in young men, which may be a presentation symptom [28, 29]. In women, sexual dysfunction will typically manifest as sexual arousal disorders [30], which are more difficult to be associated with a neurological disease. Other forms of autonomic dysfunction, such as gastrointestinal disturbances, can also be easily misattributed to other conditions. The identification of neurological symptoms may accelerate the referral to a neurologist, potentially facilitating an early diagnosis process. This underscores the importance of clinicians from different specialties being aware of this disease, as they may be the first professionals these patients meet.

It is usually reported that late onset cases are associated with a more difficult diagnostic process [15, 28]. However, the present study does not support this assertion for this specific population of sporadic patients. The origin of patients (high prevalence vs low prevalence regions) was also not associated with the delay in diagnosis. Another important related aspect, which could be of great importance, is the origin of both parents. Although it was not possible to be obtained for this study, this information should be taken into consideration by clinicians. Red flags for the identification of the disease have been previously described [29] and we propose that information about their parents should be used as a hint or red flag. This involves not only their origin but also signs and symptoms that could be indicative of ATTRv amyloidosis, even if not recognized as such. In fact, approximately 22% of the patients in our study had family members with symptoms that could be attributed to ATTRv amyloidosis, raising questions about whether clinicians are giving enough attention to the clinical history of family members.

Only three patients in our population presented with CTS, consequently, its impact on our results is minimal. Given that neuropathic pain and paresthesia are neurological symptoms, we decided to associate CTS with neurological manifestations. Due to insufficient sample sizes in each time period, we could not analyze the effects of subgroups with cardiovascular, renal, ocular, or isolated weight loss on diagnostic delay. Given the near-ubiquity of the Val30Met mutation in our population, a small number of patients presenting with cardiac manifestations was observed, precluding a specific analysis for this subpopulation.

Our study has some limitations. Firstly, it is retrospective. Additionally, the data was exclusively gathered from a single reference centre and, after applying the exclusion criteria, 24 patients were excluded, constituting approximately 18% of the initial sample. The sample predominantly consisted of V30M patients with a neuropathic phenotype. Data regarding the dates of diagnosis and/or symptom onset had to be imputed in some cases, which could have led to some degree of error when calculating diagnostic delay, with the possibility of negatively influencing this analysis. Finally, the total delay until the correct diagnosis is related not only to health care system delay, but also to how long it takes patients to see a doctor for the first time after the initial symptoms appear. However, this last aspect was not possible to be obtained, and it would have been important to truly understand if the problem lies more on clinicians who do not diagnose the disease timely, or in patients who seek for help too late in the course of their disease. Despite all this, information regarding ATTRv amyloidosis must be widely shared through the medical community and the general population.

Sporadic cases must not be overlooked. This group is particularly challenging to diagnose, leading to a more prolonged time from the onset of symptoms until diagnosis. Therefore, we must conduct more studies on misdiagnosis and predictors of diagnostic delay in these patients. This will enable us to make the necessary adjustments in our day-to-day clinical practice and improve the situation.

## Data Availability

Data will be available on reasonable request by interested researchers.
